# The Basics of Sentinel Lymph Node Biopsy: Anatomical and Pathophysiological Considerations and Clinical Aspects

**DOI:** 10.1155/2019/3415630

**Published:** 2019-07-29

**Authors:** Nasuh Utku Dogan, Selen Dogan, Giovanni Favero, Christhardt Köhler, Polat Dursun

**Affiliations:** ^1^Akdeniz University, Department of Obstetrics and Gynecology, Antalya, Turkey; ^2^TÜBİTAK Research Scholar, Ankara, Turkey; ^3^Asklepios Hospital, Department of Advanced Operative and Oncologic Gynecology, Hamburg, Germany; ^4^Başkent University, Department of Obstetrics and Gynecology, Ankara, Turkey

## Abstract

Sentinel lymph node (SLN) is the first node to receive the drainage directly from a tumor. Detection and pathological examination of the SLN is an important oncological procedure that minimizes morbidity related to extensive nodal dissection. SLN biopsy was first reported in 1960 but took approximately 40 years to come into general practice following reports of good outcomes in patients with melanoma. After many years of observation and research on its use in various malignancies SLN biopsy has become the standard surgical treatment in patients with malignant melanoma, breast, vulvar, and cervical cancers. Along with the introduction of new technologies, such as the fluorescent dyes indocyanine green (ICG) and near-infrared fluorescence (NIR), and pathologic ultrastaging, SLN detection rate has increased and false-negative rate has decreased. This literature review aimed to present an overview of the basic concepts and clinical aspects of SLN biopsy in the light of the current research.

## 1. Introduction

Sentinel lymph node (SLN) is the first lymph node to receive drainage directly from a tumor. Detection and pathologic examination of the SLN can potentially alter the extent and radicality of oncologic surgery. In other words, SLN biopsy could be considered a triage procedure. When SLN is tumor-free, systematic retroperitoneal lymphadenectomy (LND) can be omitted leading to a significant reduction in surgery-related morbidity. The SLN concept was initially proposed in 1960 and is currently considered among the most important advances in cancer therapy [1]. After many years of research on various types of malignancies SLN biopsy has become the standard of care in the treatment of melanoma, breast, vulvar, and cervical cancer, sparing many patients from the morbidity associated with ultraradical surgery. In some instances, even in pediatric patients, SLN biopsy can be a useful tool for minimizing the risks and morbidity associated with surgery [2]. The present literature review aimed to provide an overview of the basic concepts and clinical aspects of SLN biopsy, including the brief history and relevant anatomical, pathophysiological, and clinical aspects in the light of the most current scientific data.

### 1.1. What Is a Sentinel Lymph Node?

SLN is the first lymph node to which a tumor initially drains. The first SLN studies included melanoma and breast cancer patients. Currently, SLN biopsy is a routine procedure for these pathologies plus cervical and vulvar cancer as well [3–6]. SLN biopsy is based on an ordered dissemination of tumor cells from peritumoral lymphatics to the SLN, and then to more distant lymph nodes. Clinical identification of these nodes is performed via injection of numerous types of tracers, dyes, and radioisotopes into the peritumoral site depending on the type and location of the tumor. Labeled lymph nodes are surgically excised and histologically examined for the presence of disease. Identification and biopsy of the SLN can correctly indicate the status of the draining lymph node basin. In 1992, Morton was the first to identify the presence of regional lymph nodes and to map SLNs using isosulfan blue in patients with melanoma [7]. Since then, the procedure has been used for other malignancies, including breast, vulvar, and cervical cancer [8].

### 1.2. Brief History

Most cancers in humans are epithelial in nature and such neoplasms metastasize via lymphatics; therefore, the lymphatic system plays a pivotal role in a significant number of malignancies. The functions and diseases associated with the lymphatic system have been extensively studied since the seventeenth century. Virchow postulated that lymph nodes function as filters [9]. The first studies on the role of the lymphatic system in the spread of cancer cells and metastasis were performed in the 1950s [10]. The route of lymphatic dissemination was initially demonstrated in animal models using a variety of injected tracers [11].

Halsted highlighted the importance of the lymphatic system in the management of breast cancer. The researcher obtained total cure in 33% of patients using radical mastectomy and en bloc resection of breast tissue and axillary lymph nodes [12]. This aggressive surgical excision concept gained popularity in the treatment of other tumors of epithelial origin in various organs of the human body such as radical gastrectomy for gastric cancer, radical hysterectomy for cervical cancer, the Whipple procedure for pancreatic cancer, and radical skin excision for melanoma. At that time empiric beliefs substantiated the relevance of radical surgery, in terms of survival; however, increased radicality would be associated with a high morbidity rate and a decrease in quality of life (QoL). In order to overcome surgery-related morbidity innovative surgical concepts, such as SLN biopsy, were hypothesized. The first clinical use of SLN biopsy was reported by Gould in 1960 [1]. He reported that a frozen section of a normal-appearing lymph node obtained during total parotidectomy was proven to be histologically positive, which led the surgeon to perform full neck dissection. SLN biopsy was also proven to be useful in patients with penile cancer. Cabanas observed that patients with penile cancer that underwent inguinal LND inevitably experienced lymphedema [13]. This clinical observation led Cabanas to resect the very first (sentinel) lymph node that emerged directly from a tumor, so as to determine if it harbored tumor cells. According to that report, in case of a positive node it is recommended that the procedure be extended to include radical removal of the regional nodes. Cabanas showed that the SLN is the first node to which a tumor drains. Those observations provide direct evidence that tumor cells spread in an organized manner, following a predetermined anatomical pathway. This theory also proved to be correct in animal models of melanoma and mammary cancer cell lines [9]. Despite these early positive findings, it took more than 40 years for SLN biopsy to be incorporated into general oncological surgical practice [7].

Giuliano was the first to propose the use of SLN biopsy in breast cancer patients via injection of isosulfan blue into the tumor site [8]. Following the introduction of radioisotopes as tracers and gamma probes, identification of the SLN was simplified. The concept of dual mapping (use of a dye along with a radioisotope) was first described in 1992 where cutaneous lymphoscintigraphy was used prior to sentinel node biopsy in melanoma by Morton et al. from John Wayne Cancer Institute. Subsequently dual mapping in SLN biopsy was used and validated in breast cancer patients as a way to increase sensitivity and detection rate [3]. SLN biopsy in patients with early-stage breast cancer was shown to be an oncologically safe procedure in three randomized controlled trials [8, 14, 15].

## 2. The Pathophysiology of Sentinel Lymph Node Metastasis

### 2.1. The Pathophysiology of the Lymphatic System and Nodal Metastasis

The hematologic behavior of metastatic cancer has been extensively studied, but little is known regarding lymphatic metastasis [16]. It was commonly thought that lymphatic metastasis is a passive event. Nevertheless, in the light of current research, this process is known to be highly dependent on lymphangiogenesis, immunomodulation, and regulation of specific cytokines [17]. In fact, angiogenesis is the initial and most important event for lymphatic metastasis. The modern concept of angiogenesis includes remodeling of blood vessels and a complex sequence of events in the lymphatic microenvironment. The first step in lymphatic metastasis is tumoral invasion of peritumoral tissues and disruption of microvascular lymphatic channels [18, 19]. In this tumoral microenvironment there is an active interaction between tumor cells, stromal cells, and the extracellular matrix. Tumor-tumor and tumor-cell interactions are autocrine- and paracrine-mediated biological phenomena that play a central role in lymphangiogenesis. The immune system also contributes to tumorigenesis via participation of lymphocytes, macrophages, and neutrophils that secrete soluble cytokines—growth factors sustaining the inflammatory reaction and neoangiogenesis [20]. Moreover, leucocytes produce metallomatrix proteases and other enzymes that degrade intercellular adhesion molecules and the basal membrane [9].

The series of events begin when tumor cells secrete local factors that weaken cell-cell junctions and degrade the basal membrane via the assistance of proteolytic enzymes; these free malignant cells diffuse easily to the peritumoral extracellular matrix. Consequently with the help of angiogenetic factors lymphangiogenesis begins. Tumoral angiogenesis is not an orderly phenomenon [21]. Compared to normal lymphatic vessels neoangiogenic lymphatics have loose pericytic spaces allowing deliberate entry of tumor cells into lymphatic circulation [22]. The movement of tumor cells in the lymphatic microvasculature is further enhanced by an increase in interstitial fluid pressure, which is primarily the result of an increase in secretion of extracellular matrix proteins [23].

Neoangiogenesis and lymphangiogenesis begin in the SLN long before the arrival of tumor cells into these nodes. These remote effects are produced directly by vascular endothelial-like growth factors VEGF A, C, and D, all of which are secreted by primary tumors [9, 24]. VEGFs were the first identified substances [25]. These factors induce neoangiogenesis directly and play a key role in metastasis. The dynamics of tumoral VEGF secretion differ from normal physiologic processes. Hypoxia in tumor tissue induces overexpression of genes responsible for VEGF regulation [9]. Additionally extracellular matrix components such as fibrinogen and fibronectin are hypersecreted via uncontrolled production of VEGFs which further induces hypersecretion of VEGFs in a positive feedback loop. During this process VEGFs play a role similar to that of histamine but their effect is much more pronounced. This is the basic mechanism explaining the invasion of tumor cells into the lymphatic system and, to a lesser extent, into blood vessels.

Tumor cells arrive at the SLN through afferent antihilus side—namely, the subcapsular sinus. Tumor cells first invade SLN's medulla, and then the hilum. The primary mechanical forces that drive these tumoral cells to SLN are increased interstitial and stromal pressure and increased lymphatic vasculature permeability [26]. When compared to lymphatics it is more difficult for tumor cells to invade blood vessels because of the presence of an extra layer in the basal membrane which might be the reason why tumor cells invade the SLN before systemic spread occurs. Subsequently, tumor cells exit the SLN through an efferent pathway to other lymph nodes [16].

### 2.2. The Rationale for Sentinel Node Mapping

SLN biopsy should be used in patients with malignancy following lymphatic spread rather than the hematogenous route. The rationale for this is that it significantly decreases morbidity without jeopardizing oncological outcome [27]. SLN biopsy was conceived as a minimally invasive alternative to systematic lymphadenectomy that does not negatively affect staging accuracy. The most important aspect of SLN biopsy is appropriate selection of patients that will benefit from this minimally invasive procedure. Although surgical complications do occur in association with SLN biopsy this is less common as compared to complete lymphonodal dissection (LND) [28]. The subsequent incorporation of ultrastaging into the histological analysis of the SLN improved the ability to identify small-volume disease unlikely to be detected via conventional hematoxylin-eosin (H&E) staining [3]. With the advent of ultrastaging new terms such as micrometastasis and isolated tumor cells came into clinical use. This terminology is currently used for substaging of certain tumors, despite the fact that their oncological relevance, in terms of recurrence and overall survival, is unclear [29]. Ultrastaging of SLN biopsy specimens consists of 3 related steps: serial sectioning, immunohistochemical analysis (IHC), and reverse-transcriptase polymerase chain reaction (RT-PCR). As the number of slices increases, errors related to sampling are significantly reduced. An advantage of SLN biopsy is a decrease in the number of harvested lymph nodes, which affords more time for a specified lymph node, increasing the chance of serial sectioning. MART-1 and S-100 IHC staining for melanoma and cytokeratin for breast and colon cancer increase the potential for detecting even isolated tumor cells [30]; however, false-positive staining of other cell types, such as dendritic leukocytes stained with S-100 and plasma cells stained with cytokeratin, can result in erroneous findings and misdiagnosis [31].

## 3. Clinical Aspects of Sentinel Lymph Node Biopsy

### 3.1. The Current Status of Sentinel Lymph Node Studies in Breast Cancer and Melanoma

#### 3.1.1. Breast Cancer

Axillary lymph node status is the most important prognostic factor for early-stage breast cancer [3]. In current practice SLN biopsy is considered the gold-standard surgical method for breast cancer nodal staging. It is a safe procedure with a low false-negative rate and low morbidity. The orderly spread of breast carcinoma theory came into question with publication of the prospective NSABP B-04 Trial in which it was reported that the addition of axillary LND to mastectomy had no effect on disease-free survival or overall survival [32]. Those findings indicate that the disease might already be systemic when it disseminates to regional lymph nodes. Nevertheless, regional lymph node status is critically important for precisely tailoring adjuvant treatment and evaluating prognosis. Among patients with a positive SLN biopsy specimen, the disease is located only in the SLN in 70% of cases, most commonly as one microscopically positive LN [8]. In the past, when SLN biopsy findings were positive for metastasis, complementary LND was performed, whereas the findings reported in the ACOSOG Z11 Study resulted in widespread change in the treatment of breast cancer [33]. That study observed that full axillary LND in patients with positive SLN biopsy had no survival benefit over patients that did not undergo axillary dissection. Recently long-term follow-up of the same study (ACOSOG Z11) was published. At a median follow-up of nearly 10 years, as expected, recurrence free survival and incidence of nodal recurrences were all comparable in two groups [34].

With the advent of ultrastaging, the accuracy of SLN biopsy has increased. Earlier false-negative cases in which small-volume disease had not been detected using traditional pathology techniques can now be considered true-positive cases [3]. It is obvious that ultrastaging facilitates more accurate detection of metastasis. With conventional lymph node pathologic assessment one tumor cell can be detected among one million normal lymphocytes, whereas ultrastaging analysis can detect one tumor among 10 million normal lymphocytes; therefore false-negative rate in SLN biopsy specimens is decreased considerably. On the other hand previously undetected low-volume disease—namely, micrometastasis and isolated tumor cells—can now be identified with greater frequency but there are a number of unresolved questions regarding the clinical significance of these findings [35]. The MIRROR Study reported that administration of adjuvant systemic therapy in patients with micrometastatic breast cancer resulted in significantly better survival [35]. Likewise, the NSABP B-32 Trial showed that occult metastasis was an independent risk factor for breast cancer relapse [36]. Moreover extracapsular involvement in the SLN was proven to be an independent marker for involvement of nonsentinel lymph nodes and a decrease in overall survival [37].

The performance of SLN depends significantly on surgeon experience. SLN biopsy performed using dye and lymphoscintigraphy increases the detection rate; however, an important meta-analysis showed that the false-negative rate associated with use of only one tracer was similar to that associated with the combination of dye and lymphoscintigraphy [38]. Currently the gold standard for SLN biopsy is lymphoscintigraphy with technetium (Tc-99m) [30]. Generally Tc-99m is injected into the ipsilateral subareolar plexus. There are logistical problems associated with acquiring radiocolloids; therefore, researchers are looking for alternative markers, such as indocyanine green (ICG). Research has shown that Tc-99m is better than ICG for preoperative identification of an SLN, but when skin incision is performed ICG is also an excellent marker. Currently Tc-99m maintains some advantages over ICG primarily because it is easier to follow the route of lymphatic drainage and to detect hotspots before a skin incision is made [39]. Another promising agent is Tc-99m-tilmanocept, a new CD206 receptor-targeted marker for SLN mapping in patients with head and neck cancer. Tc-99m-tilmanocept molecules are so small that they can readily enter reticuloendothelial cells via CD206 mannose-binding receptors in SLNs. This marker has a higher SLN detection rate and negative predictive value than other radiocolloids [40].

The use of SLN biopsy in breast cancer decreases the morbidity related to LND. This also includes shorter duration of drain use, hospitalization, and the time to resumption of normal daily activities [27]. Moreover, all QoL parameters following SLN biopsy were better than those following systematic nodal dissection. The most important consequence of complete axillary dissection is lymphedema which occurs in nearly half of the patients undergoing full lymphadenectomy. With the use of the SLN biopsy the frequency of this negative event can be decreased to 1%-2%.

Another important consideration is the feasibility of SLN biopsy following neoadjuvant chemotherapy (NACT). For a long time complete axillary LND was routinely performed in patients taking NACT as the first-line treatment [41]. However recent research has cast doubt on the validity of this routine treatment leading to studies on the role of SLN biopsy—even after NACT [42]. The optimal sequencing of SLN biopsy—before or after NACT—is difficult to precisely determine but both have advantages and disadvantages. The primary advantage of SLN biopsy after NACT is its ability to detect the patients who have the best results after the treatment with negative SLN [43]. In a way, SLN biopsy is a prognostic factor when performed following NACT. In contrast, the positive SLN detection rate is higher when SLN biopsy is performed prior to NACT [41]. The detection rate when SLN biopsy is performed after NACT ranges between 85% and 95% in large series [41, 44]. Moreover more patients would have negative SLN after NACT. In other words, these patients would be spared from undergoing more aggressive axillary dissection when SLN biopsy is performed after NACT [41]. This approach is useful and safe for clinically node-negative patients. The role of SLN biopsy after NACT for clinically node-positive patients is controversial. In fact the logic behind the use of SLN biopsy in clinically node-positive patients is the conversion of node-positive patients into node-negative patients with the use of chemotherapy as first-line treatment. However a high false-positive rate and lack of long-term follow-up of node positive patients currently render this approach unattractive, pending the publication of findings from ongoing long-term trials [45].

#### 3.1.2. Malignant Melanoma

As with other cancers the most common site of initial metastasis in patients with melanoma is the regional lymph nodes. The status of these nodes is the most important factor associated with survival and the risk of recurrence [4]. Lymph node positivity decreases survival significantly. However approximately 80% of patients do not have lymph node involvement and will not benefit from full LND which would only be performed for diagnostic purposes [46]. SLN biopsy can spare these patients from such a risky operation without any therapeutic effect. SLN biopsy is proven to be an accurate diagnostic tool and is considered to be the standard of care for intermediate-thickness melanoma (1.01-4.0 mm) [47]. In contrast the use of SLN biopsy for tumors <1 mm or >4 mm, as well as for desmoplastic tumors, remains controversial [4]. There are a number of randomized controlled trials on the role of SLN biopsy in patients with malignant melanoma. The Multicenter Selective Lymphadenectomy Trial (MSLT-I) evaluated the role and oncological effect of SLN biopsy for primary melanoma, reporting that the technique is feasible for intermediate-thickness melanoma [48]. SLN biopsy increased melanoma-specific survival in patients with a positive LN, as compared to the observation arm. In cases of SLN positivity full lymphadenectomy was performed. The reported survival benefit might be related to the detection of occult disease and the therapeutic effect of nodal metastasis debulking. Recently results of MSLT-II phase III trial were also published. In this study in case of a positive SLN, completion of lymph node dissection was compared to observation without further intervention. As expected, complete dissection did not improve the melanoma specific overall survival although there was a benefit with respect to regional disease control [49]. The Sunbelt Melanoma Trial is another important study on the effect of interferon in SLN-positive patients that consequently undergo complementary regional LND. The value of isolated positive marker SLN (negative in H&E and also IHC) and the effect of additional treatment were also analyzed. However there was no survival benefit of treatment versus observation [50]. Moreover the results of multicenter DeCOG-SLT trial support the omission of complete lymphadenectomy when SLN biopsy is proved to be positive. In the final analysis of DeCOG-SLT study, in SLN positive cases, there was no difference in five-year distant metastasis-free and overall survial in 483 patients with immediate completion of remaining lymph node basins compared to observation. Now, based on the result of MSLT-II and DeCog multicenter trials, the standard of care for intermediate thickness melanoma patients with positive sentinel node is observation [51].

For melanomas <1 mm thick routine use of SLN biopsy is not justified and is not recommended. Yet in certain circumstances such as the presence of tumor ulceration and invasion exceeding 0.76 mm the accuracy of SLN biopsy increases and could be considered as an alternative approach to systematic LND [47]. Because regional LN and distant metastasis rates are high, the use of SLN biopsy is controversial in patients with melanomas >4 mm thick. The most common complications in melanoma patients undergoing SLN biopsy are wound infection, hematoma, pain, numbness in the surgical field, and lymphedema. Rarely, allergic reactions occur. In the MSLT-I Trial 10% of patients that underwent SLN biopsy had complications versus 33% of patients undergoing full LND [48].

### 3.2. Sentinel Lymph Node Biopsy for Gynecologic Cancers

#### 3.2.1. Vulvar Cancer

Although vulvar cancer is a rare genital malignancy, its incidence has been increasing particularly in northern Europe and the US since the 1990s [52]. Vulvar malignancies frequently affect elderly women with multiple comorbidities and classically the standard oncologic treatment consists of radical vulvectomy and inguinal LND. Inguinal LND is an operation with a high morbidity and about 65% of patients have such complications as wound breakdown, lymphocele formation, and most importantly lymphedema [53]. Approximately a third of patients that undergo inguinal LND are proven to be positive and the surgery exposes a significant number of those patients to serious complications [54]. On the other hand the most important prognostic factor in patients with vulvar cancer is nodal status and lymph node recurrence is inevitably lethal [55]. In such cases the advantages of conservative surgery should be balanced against the risks of overlooking a metastatic lymph node. The GROINSS-V study (Groningen International Study on Sentinel Nodes in Vulvar Cancer) has resulted in some important shifts in the paradigm regarding the surgical treatment of vulva cancer [55]. That observational study on early-stage vulva cancer included patients with tumors <4 cm that underwent SLN biopsy only or underwent complete inguinal dissection; the recurrence rate was similar in both groups, with a 5% false-negative rate. More importantly, complications associated with radical nodal dissection decreased significantly. Presently, SLN biopsy is standard treatment in women with tumors <4 cm. In this subgroup of patients the false-negative rate is acceptable (around 3%). The best detection rates and lowest false-negative rates are obtained when combined methods (i.e., radiocolloid and any dye method) are utilized.

Many studies have evaluated SLN biopsy in patients with vulvar cancer but the studies' methodologies were inconsistent and consequently such methodologic differences in these studies led to widespread debate among oncologists. In fact SLN biopsy is most precise in patients with tumors <4 cm that are located 2 cm from the midline and are obtained via combined techniques (radiocolloid and patent blue) [5, 56]. In some instances such as previous excision of the primary tumor and following administration of neoadjuvant therapies (chemotherapy and/ or radiotherapy) the accuracy of SLN biopsy can be significantly decreased [56]. There are no data regarding the use of SLN biopsy after administration of NACT and patients that have received such treatment should be recommended full inguinal LND. Patients that underwent excisional biopsy prior to primary surgery had comparable detection rates and false-negative rates when SLN biopsy was used [57]. Although these earlier studies included small patient populations, excisional biopsy before surgery should not be a contraindication to the use of SLN biopsy in this setting [58]. Additionally what remains to be discerned are the optimal treatment strategy for micrometastasis in SLNs identified via ultrastaging and the role of completion of LND in cases of macroscopic disease. These questions might be answered by the currently ongoing GROINSS-V II Study (GOG 270) [59]. It is hoped that data provided by this study will definitely show if a more conservative approach (chemoradiotherapy rather than complementary inguinofemoral dissection)—even in SLN-positive patients—is oncologically safe or not.

The SLN detection rate and the sensitivity of SLN biopsy both increase along with surgeon experience. The learning curve for SLN biopsy is considered to be approximately 10 cases in which the first step constitutes SLN biopsy followed by systematic LND [56]. Additionally it is highly recommended that SLN biopsy and local surgical treatment of vulva cancer be performed in reference centers where a significant number of vulvar cancer patients are treated on yearly basis. With respect to cost effectiveness, despite the fact that SLN biopsy requires additional pathology and nuclear medicine techniques, the procedure is more cost effective than complete LND (~$4000 per patient) due to a low complication rate that is associated with lower postoperative medical costs [60].

#### 3.2.2. Cervical Cancer

Although SLN biopsy is not currently the standard of care worldwide in patients with cervical cancer, it can be safely utilized in women with early-stage disease and tumors <2 cm. Indeed, the latest version of the NCCN Cervical Cancer Guidelines (version 3.2019) considers SLN biopsy in patients with early-stage cervical cancer <2 cm an alternative to complete pelvic LND [6]. In earlier studies the SLN detection rate varies between 50% and 100%, depending on the technique used [61]. The SLN detection rate increases with the use of combined techniques, namely, patent blue and TC-99m, together with preoperative lymphoscintigraphy. With the introduction of ICG and NIR (near-infrared fluorescence) technology, the SLN detection rate has increased considerably. Buda et al. observed in 2015 that in 88% of patients bilateral SLN can be detected with this technique [62]. Moreover, according to the SENTICOL Study the sensitivity of SLN biopsy when combined with ultrastaging is 92% and can increase to 100% when bilateral SLN is identified [63]. Ultrastaging facilitates detection of low-volume disease in a larger number of patients. As with other malignancies, the oncological significance of low-volume tumors—in particular isolated tumor cells—in patients with cervical cancer is obscure. Nevertheless, micrometastasis (tumors size 0.2-2 mm) is an important prognostic factor and should be considered when assessing the necessity of adjuvant treatment [64].

Another promising marker of SLNs is HPV-E6/E7-mRNA. Recently, Köhler et al. evaluated the role of HPV-E6/E7-mRNA detection in SLNs in patients with cervical cancer undergoing laparoscopic LND [65]. In total they excised 125 SLNs of which 10 were tumor-positive and proven to be positive for HPV-mRNA. Additionally 4 LNs were tumor-free but positive for HPV-mRNA. The commercially available rapid HPV-mRNA test is a promising method for detecting positive SLNs and in the future might replace use of frozen sections.

Unlike axillary LND for breast cancer, whether or not systematic LND should be performed in cervical cancer patients with SLN positivity remains unclear. The current approach in patients with early-stage cervical cancer is radical hysterectomy or fertility-sparing surgery as radical trachelectomy when the SLN is tumor-free [66, 67]; however, when the SLN is positive, radical hysterectomy or trachelectomy can be abandoned in favor of primary radiochemotherapy and extended LND. The rationale behind this approach is to spare the patient from the morbidity associated with two treatment modalities—surgery and postoperative chemoradiation. It is yet to be proven by randomized controlled trials that abandoning radical hysterectomy in patients with a positive LN is beneficial; therefore, the use of SLN biopsy in patients with cervical cancer can potentially change how oncological surgical treatment is performed. It is very important to maintain a strict protocol and any suspicious LN should be excised. Moreover, when an SLN is not detected on either side of the pelvis site-specific (ipsilateral) complete LND must be performed.

#### 3.2.3. Endometrial Cancer

Endometrial cancer (EC) is the most common gynecologic cancer and is the sixth most common malignancy in women worldwide [68, 69]. In addition, lymph node metastasis is the most important prognostic factor in cases of endometrial cancer [70]. Approximately 10% of all patients with apparently early-stage EC will be diagnosed with lymph node metastasis. In two randomized trials LND for early-stage EC did not have any survival benefit, but in patients with high-risk disease the retrospective data supports use of systematic retroperitoneal nodal dissection [71–73]. Currently, LND is the most accurate method for assessment of lymph node status. Although surgical experience reduces major intraoperative adverse events, postoperative complications, such as deep venous thrombosis, potentially lethal pulmonary emboli, chylous ascites, lymphoceles, and lymphedema, are of great concern. SLN biopsy is a promising tool for reducing surgery-related morbidity. In 1996 Burke et al. published the first series of EC patients to undergo SLN mapping; SLN mapping was performed using subserosal injection of blue dye into the uterine fundus [74]. Subsequently, numerous studies on the use of other tracer injection variations flooded the literature; yet, there remains no consensus regarding the optimal technique. Superficial cervical, deep cervical, and hysteroscopic subtumoral administration have been tested with inconsistent results. The detection rates are similar across methods; however, mapping of the para-aortic region is better when using fundal and deep cervical tracer injection [5].

One of the most commonly used protocols in EC SLN studies is that suggested by Barklin et al. in 2012; accordingly, markers should be injected superficially and deeply in the uterine cervix, and the obtained sentinel nodes should routinely be subjected to ultrastaging [75]. Along with institutional incorporation of this predetermined protocol, the sensitivity and negative predictive values increased and the false-positive rate decreased significantly. Additionally, the number of systematic LNDs performed decreased considerably, as well as the duration of the procedures and number of harvested lymph nodes. In low-risk EC patients LND could be omitted, with respect to preoperative and intraoperative findings; however, this approach is not always possible and is not readily available, because ≤25% of tumors preoperatively designated as grade 1 and about 20% of tumors classified as grade 1-2 carcinoma intraoperatively based on frozen analysis could be upgraded via final pathology [76]. Moreover, intraoperative frozen section analysis is not always a reliable parameter, especially when an experienced gynecopathologist is not available. Due to these issues, SLN mapping can aid in the identification of patients requiring full LND [75].

After a reasonable learning curve, a high rate of SLN detection can be achieved. Use of an SLN mapping algorithm facilitates side-specific evaluation of pelvic lymph nodes. When a unilateral pelvic SLN cannot be detected, site-specific full LND should be performed on that side. Another important consideration is mapping of para-aortic lymph-basins. Mapping of the region above the common iliac artery is not as reliable as mapping pelvic lymph nodes [77]. Hysteroscopic injection is the best method to use for identifying higher lymph nodes, but this procedure is quite challenging. Moreover, positive para-aortic lymph nodes are most likely located above the inferior mesenteric artery (particularly when pelvic nodes are negative) and currently para-aortic mapping in early-stage EC is less clearly defined.

More recent studies have confirmed the feasibility and accuracy of NIR fluorescence imaging combined with ICG injection [5, 78]; researchers reported better detection rates than those obtained using radionucleotide (with preoperative lymphoscintigraphy). The fluorescent technique is more expensive due to the special equipment needed to visualize ICG and, therefore, it is not widely utilized. Independent of the technique employed for SLN identification, execution of conventional pathology along with advanced IHC staining for cytokeratin (ultrastaging) is strongly recommended, so as to minimize the false-negative rates [79]; however, as in cases of breast and cervical cancer, the presence of low-volume disease (micrometastasis and isolated tumor cells) in the lymph nodes requires further scientific clarification.

### 3.3. Sentinel Lymph Node Biopsy in Other Cancers

SLN biopsy in patients with colorectal cancer is not widely used. In cases of early-stage colorectal cancer, particularly pedunculated polyps, endoscopic resection of the effected part of the colon is sufficient and the role of LND, especially in patients with comorbidities, is controversial. Moreover, the SLN detection rate and the negative predictive value are not precise enough to justify SLN biopsy, probably due to skip metastasis [80]. In patients with urooncologic disease, except penile cancer, the use of SLN biopsy has also not gained wide acceptance because of limitations associated with multiple primary lymphatic drainage of the bladder and prostate; hence, extended LND remains the standard of care for these malignancies [81].

## 4. Future Perspectives

SLN biopsy is the standard of care for melanoma, and breast, vulvar, and cervical cancer. We expect that in the near future the procedure will be routinely used for endometrial malignancies. As clinician experience with ICG and NIR technology improves, the detection rate and accuracy will also improve. Another rapidly developing field of medical science is the use of nanoparticles. A recent study evaluated the use of a novel technology, gold-silica surface-enhanced resonance Raman spectroscopy (SERRS), reporting promising results. Moreover, with the advent of nanoparticles radioactivity associated with the use of radionucleotides (Tc-99m) will be no longer a concern [82]. The management of patients with a positive SLN remains controversial. In patients with breast cancer, it has been determined that complementary LND should be omitted, because the disease is so far systemic and patients will benefit more from adjuvant oncological treatment than radical nodal dissection. In terms of patients with vulvar cancer, the results of the GROINSS-V II Study will help to determine the safety of replacing complete inguinal femoral lymphadenectomy with adjuvant radiotherapy in those with early-stage disease and SLN metastases ≤2 mm.

## 5. Conclusion

It has been more than two decades since the first introduction of SLN procedure into medicine. Since then, our improved understanding of the lymphatic system and lymphangiogenesis led to a new era in the treatment of malignancies with lymphatic spread. The routine use of SLN biopsy could significantly decrease surgery-related morbidity, without jeopardizing oncological outcome. With the introduction of routine SLN biopsy for the surgical treatment of other malignancies, improvement in patient QoL is expected. More studies are needed to test the use of new agents and technologies in conjunction with SLN biopsy.
